# Seroprevalence, incidence estimates, and environmental risk factors for dengue, chikungunya, and Zika infection amongst children living in informal urban settlements in Indonesia and Fiji

**DOI:** 10.21203/rs.3.rs-5141509/v1

**Published:** 2024-11-13

**Authors:** Joelle I. Rosser, John J. Openshaw, Audrie Lin, Ruzka R. Taruc, Autiko Tela, Nursehang Tamodding, Nurul Pausi Emelia Abdullah, Murni Amiruddin, Esra Buyukcangaz, S. Fiona Barker, Amelia Turagabeci, Ansariadi Ansariadi, Karin Leder, Isra Wahid

**Affiliations:** Stanford University; Stanford University; University of California Santa Cruz; Indonesia Team, Revitalizing Informal Settlements and their Environments (RISE); Fiji National University; Universitas Hasanuddin; Universitas Hasanuddin; Universitas Hasanuddin; Stanford University; Monash University; Fiji National University; Universitas Hasanuddin; Monash University; Universitas Hasanuddin

**Keywords:** Aedes, trash, waste, arboviruses, vector borne diseases, dengue, built environment

## Abstract

**Background:**

The burden of *Aedes aegypti*-transmitted viruses such as dengue, chikungunya, and Zika are increasing globally, fueled by urbanization and climate change, with some of the highest current rates of transmission in Asia. Local factors in the built environment have the potential to exacerbate or mitigate transmission.

**Methods:**

In 24 informal urban settlements in Makassar, Indonesia and Suva, Fiji, we tested children under 5 years old for evidence of prior infection with dengue, chikungunya, and Zika viruses by IgG serology. We used a catalytic model using seroprevalence and mean age to estimate annual incidence of dengue in each country. We also conducted detailed questionnaires to evaluate environmental risk factors for a positive serology result. Dengue risk factors were evaluated for individual children by univariate and multivariable logistic regression accounting for settlement as a flxed effect. Trash and flooding were additionally evaluated as dengue risk factors at the settlement level by univariate linear regression.

**Results:**

In Fiji and Indonesia respectively, 46% and 33% of children under 5 years old were seropositive for dengue, 3% and 3% for chikungunya, and 9% and 2% for Zika. In Indonesia, children living in a household where trash is routinely collected and removed were signiflcantly less likely to be dengue seropositive in both unadjusted and adjusted models [adjusted model: OR 0.3 (95% CI: 0.1–0.8)]. In Indonesia, settlements with a higher proportion of households reporting flooding also had lower dengue rates (slope = 0.44; p-value: <0.05).

**Conclusions:**

Household trash collection and community flood management are important targets for interventions to mitigate the increasing risk of *Aedes aegypti*-transmitted viruses.

## Background

Warming temperatures and extreme weather events are expanding the range and availability of suitable habitats for *Aedes aegypti*, the primary mosquito vector for dengue, chikungunya, and Zika viruses[1–6]. Dengue virus is the most common arbovirus globally, has increased exponentially over the last several decades[7], and causes an acute febrile illness with clinical presentations ranging from asymptomatic to life-threatening hemorrhage and shock[8]. While less prevalent than dengue, chikungunya and Zika viruses have also emerged as global problems over recent decades and can cause long term morbidity[8–12].

As the climate continues to change, generating reliable estimates of infection rates for *Ae. aegypti* transmitted viral infections is essential to monitoring changing transmission dynamics and generating models to forecast future risk. Current estimates of recent disease transmission are limited by a reliance on acute febrile surveillance and cross-sectional serology studies in the general population. Acute febrile surveillance underreports the true burden of disease, only tracking cases that come to the hospital and receive a correct diagnosis. Dengue, chikungunya, and Zika infections all have non-speciflc clinical presentations; and diagnostic tests for infections are not readily available in many medical systems and have a limited window for detection. Furthermore, surveillance reporting requirements and resources can vary between locations and over time. An alternative to acute febrile surveillance is estimating disease burden with cross-sectional serology studies. Seroprevalence studies are also limited in that a positive serology indicates any past infection, not just recent infection, and antibody levels wane over time. However, in places with a high incidence of disease, serology studies performed in young children can overcome these limitations and provide insights into recent disease incidence.

While climate change may be fueling the spread of *Ae. aegypti*-transmitted viruses, local environmental factors can also affect an individual’s risk of exposure. *Ae. aegypti* mosquitoes breed in small containers of fresh water, including water storage containers, trash, discarded tires, and gutters on houses that flll with rainwater[13–25]. Local conditions, including temperature, humidity, and air circulation, also affect where the adult mosquitoes reside[5, 26, 27]. The role of the built environment, such as housing construction[24, 27–29] and landscaping[27], in modulating dengue risk is beginning to be recognized. As climate change exacerbates *Ae. aegypti*-transmitted viruses, there is a critical need for better understanding modiflable features of the built environment that attenuate transmission risk and can be targets for local interventions.

Informal urban settlements in Indonesia and Fiji have known high rates of dengue infection[1, 30–32]. The hot, humid climate is highly suitable for the *Ae. aegypti* lifecycle and virus incubation[33–35]. Fluctuations between drought and flooding in the region[36, 37] and inadequate water infrastructure[38] in the settlements results in pooling of rainwater during heavy rains and storage of water during dry periods, both providing breeding grounds for *Ae. aegypti* mosquitoes. Inadequate trash management can additionally provide containers for mosquito oviposition[13, 27].

The objectives of this study are to measure seroprevalence and estimate the incidence of dengue, chikungunya, and Zika infections in young children living in informal urban settlements in Indonesia and Fiji. This study also aims to evaluate local environmental risk factors for *Ae. aegypti* – transmitted arbovirus infections.

## Methods

### Study Population

The Revitalizing Informal Settlements and their Environment (RISE) study was conducted in 24 informal urban settlements in Makassar, Indonesia and Suva, Fiji with enrollment and study procedures previously described[39]. This study involved questionnaires and biological sampling of children between the ages of 6 months to 5 years old living in the RISE sites in 2018 and 2019 who were enrolled in the study and whose parents consented for their participation.

### Questionnaires

Baseline questionnaires were used to assess household and individual demographic information and environmental exposures that were hypothesized to be predictive of dengue, chikungunya, and Zika seropositivity.

### Serology testing

We performed dengue, chikungunya, and Zika virus serology testing on serum samples collected from children under 5 years old enrolled in the RISE study who underwent sampling in 2018 in Indonesia and in 2019 in both Indonesia and Fiji. Serum samples were stored in Sarstedt screw cap tubes at −80°C for four years prior to serology testing. Serology testing was performed in duplicate using Abcam IgG ELISA’s kits to evaluate for evidence of prior exposure to dengue, chikungunya, and Zika viruses. Duplicate positive, negative, and cut-off controls were used on each plate. Following Abcam kit protocols, antibody titers were converted into standard units based on average cut-off values and all samples with titers greater than 10 standard units were considered positive. Seropositivity rates are reported for each arbovirus and for the proportion of children with evidence of multiple prior infections.

### Incidence estimates

Catalytic models estimate the force of primary infection, or incidence rate, using seroprevalence data in diseases where seroprevalence is a marker of any past infection and indicates lifelong immunity[40, 41]. Dengue infection, particularly in young children, meets these criteria and catalytic models have previously been used to estimate dengue incidence[42]. Using a catalytic model, we estimated dengue incidence in each country assuming a constant force of infection over time where by *incidence = 1–(1–seroprevalence)^(1/ mean years of exposure)*.

### Risk factor analysis

Baseline demographic and environmental risk factors for dengue infection in the children enrolled in the RISE study were evaluated by a univariate logistic regression model and a multivariable logistic regression accounting for settlement as a flxed effect and individual characteristics thought to be plausible risk factors for dengue as random effects. For the two breastfeeding questions, “breastfeeding currently” was retained in the multivariable model since it was signiflcant in the univariate model in Fiji; however, a sensitivity analysis was also performed which replaced “breastfed in the past 3 months” in the model, which did not change the flndings.

As a further exploration of the potential impact of household variables that might have an impact on the surrounding settlement arbovirus exposure risk – namely flooding and trash collection - we also conducted univariate linear regression at the settlement level to evaluate whether the proportion of households reporting flooding and trash collection were predictive of dengue seropositivity across the settlement. This settlement analysis was restricted to settlements with at least 10 children tested and multivariable regression was not conducted given the small sample size.

All risk factor analyses were conducted to evaluate risk of dengue infection. Given the overall low chikungunya and Zika seroprevalence and the fact that the majority of individuals with evidence of either of these two infections were seropositive for dengue, risk factor assessment was not conducted for these other viruses. All analyses were performed in R version 2023.06.1.

#### Ethics:

Ethics review and approval was provided by participating universities and local IRBs, including: Monash University Human Research Ethics Committee (Melbourne, Australia; project ID 35903), Ministry of Research, Technology and Higher Education Ethics Committee of Medical Research at the Faculty of Medicine, Universitas Hasanuddin (Makassar, Indonesia; protocol UH18020110), and Fiji National University College Human Health Research Ethics Committee (CHREC ID 137.19). This trial is registered with the Australian and New Zealand Clinical Trials Registry (ACTRN12618000633280; https://www.anzctr.org.au/).

## Results

### Demographic Characteristics

A total of 191 children in Fiji and 181 children in Indonesia were included in the study. Overall, the mean age of children at the time of serum sample collection was 3.3 years old and male children comprised 61% of the study population. Breastfeeding, household trash collection, having a household member who grows plants, and porous building materials for household construction was more commonly reported amongst participants in Fiji than Indonesia. In contrast, flooding in or around the house was more commonly reported in Indonesia. (Table 1)

### Seropositivity

Dengue IgG seropositivity was high in both countries, with 46% of children in Fiji and 33% of children in Indonesia demonstrating evidence of prior dengue exposure. Zika seropositivity was higher in Fiji than Indonesia. Chikungunya seropositivity was low but detectable in both countries. A total of 19 children (10%) in Fiji and 7 children (3%) in Indonesia were seropositive for more than one arbovirus. (Table 2)

Dengue seropositivity overall increased with age, with the exception of the 0.5 to < 1 year olds which showed a relatively high seropositivity but had very low numbers of participants. By the age of 4 to 5 years old, 71% and 51% of the children in Fiji and Indonesia respectively had been infected with dengue (Table 3). Using a catalytic model, we estimated an annual incidence rate of 18% in Fiji and 11% in Indonesia. Although seroprevalence rates of chikungunya and Zika were too low to model incidence, seropositivity was found in multiple age groups.

#### Demographic and environmental risk factors for dengue:

Age was a signiflcant predictor of dengue serostatus amongst children in Fiji and Indonesia in both unadjusted and adjusted models [Adjusted models - Fiji: OR 4.0 (95% CI: 2.5–6.3); Indonesia: OR 2.2 (95% CI: 1.4–3.6)]. In Indonesia, children living in a household with trash collection were signiflcantly less likely to be dengue seropositive in both unadjusted and adjusted models [Adjusted model OR 0.3 (95% CI: 0.1–0.8)]. Living in a house made of porous flooring material was also protective against dengue exposure amongst children in Indonesia, although this was only signiflcant in the unadjusted model [OR 0.4 (95% CI: 0.2–0.9)]. In Fiji, children who were currently breastfeeding at the time of serum sampling were less likely to be seropositive for dengue in the unadjusted model [OR 0.3 (95% CI: 0.1–0.7)], but this did not remain statistically signiflcant in the adjusted model accounting for age. (Table 4) Household flooding was not a signiflcant predictor of individual dengue seropositivity in Indonesia or Fiji; however, in Indonesia, settlements with a higher proportion of households reporting flooding had lower rates of dengue. (Figure)

Settlements in Indonesia with a higher percentage of houses that reported flooding in or around their house had signiflcantly lower dengue seropositivity rates, suggestive that flooding could reduce breeding habitats.

Settlements in both Fiji and Indonesia with a higher percentage of houses reporting trash collection seem to have lower dengue seropositivity rates, consistent with the individual level analysis, but this was not found to be signiflcant at the settlement level. In Fiji, trash collection was found to be high across most settlements, making any potential correlation to dengue risk difficult to ascertain.

## Discussion

Our study found a high prevalence of dengue in Fiji and Indonesia, with over half of children in each country having had an infection by the age of flve years old. Although chikungunya and Zika exposure was lower, a seroprevalence of 2–9% in such young children suggests ongoing transmission of these other *Ae. aegypti* – transmitted viruses in these countries as well.

Our dengue seroprevalence estimates are similar to those found in a 2014 study of urban children across Indonesia[32], and in South Sulawesi in particular[43], corroborating the high rates of dengue in Indonesia. Although dengue circulation in Fiji is well established with many known outbreaks over the years, dengue seroprevalence and incidence data across Fiji is limited[44, 45]. Our study is one of the few studies providing such estimates and showed high circulation of dengue in Suva, the capital city located in the Central District of the Island of Vitu Levi. Our study also showed a steady increase in seropositivity for each year of age in children under 5 years old in both countries, indicating high levels of non-epidemic transmission.

Other studies in Indonesia and Fiji looking at demographic or environmental risk factors have not identifled signiflcant risk factors for dengue seropositive test results[32, 44]. Our study focused on environmental features of the built environment that would be expected to increase dengue exposure and looked at the youngest children as they acquire their flrst dengue infections, thus giving a potentially unique window into risk factors in places with very high levels of risk.

Trash being collected and removed from the household in our cohort in Indonesia was a signiflcant protective factor against dengue infection in the individual level analysis. In the secondary analysis at the settlement level, we observe a similar correlation between settlement trash collection and settlement dengue risk. Although the settlement level analysis was not found to be statistically signiflcantly, we suspect this is due to inadequate statistical power with this small sample size or that household practices are a stronger driver of risk given the relatively short flight range of *Ae. aegypti.* We did not flnd a signiflcant association between trash collection and dengue in the Fiji cohort in either the individual or community level analyses. In Fiji, the higher overall dengue risk may overwhelm such individual environmental risk; furthermore, trash collection was very common (83%) in Fiji, diminishing our power to detect a difference in dengue exposure in this cohort. *Ae. aegypti* breed in small containers of water[13, 14, 18, 20] and have a relatively short range of about 100 meters[46]. Our flndings in Indonesia corroborate other studies demonstrating *Ae. aegypti* breeding in trash fllled with rainwater [13, 27, 47–49] and implicate household trash disposal practices as a dengue exposure risk. Our study adds to the existing literature not only by showing this direct association between household trash disposal practices and individual disease risk but also by highlighting the risk posed by inadequate trash collection in informal settlements. Because they are not legally recognized neighborhoods, informal settlements are often excluded from government trash collection programs, particularly in Makassar, Indonesia. Our study indicates that household trash removal could decrease disease exposure and endorses the inclusion of informal settlements in regular government trash collection programs.

The impact of flooding on dengue risk is complex and remains poorly understood. While dengue outbreaks following large floods frequently make news headlines[50–52], it is unclear whether these outbreaks are due to the floods themselves, heavy rainfall regardless of flooding, or an extended duration of pooled water as floodwaters recede. Other studies have proposed that while heavy rainfall fllls up potential *Ae. aegypti* breeding grounds with water, severe flooding may in fact flush out those small containers holding water and *Ae. aegypti* larva, or wash away trash containers from the area, thereby decreasing risk[35, 53]. Our analysis at the individual level did not show flooding to signiflcantly increase or decrease the risk of dengue infection in the child cohorts in either country, perhaps reflecting this complexity. Interestingly though, when we considered flooding to be a settlement risk and evaluated the relationship between the degree of settlement flood exposure and settlement dengue prevalence, in Indonesia we found that settlements with more households experiencing flooding had fewer children with positive dengue serologies. Our flndings support the hypothesis that flooding may flush out *Ae. aegypti* larva or reduce trash that support those larva[35], although given our small sample size, these flndings should be interpreted cautiously. As extreme flooding increases with climate change, further studies are needed to better understand how flood microclimates affect *Ae. aegypti* proliferation and consequent disease risk.

Finally, our study hints at the possibility that housing construction made of porous materials could be protective against dengue exposure. We had initially hypothesized that porous housing materials might let mosquitoes into the house which would increase risk. However porous housing construction could allow for increased ventilation and indoor lower temperatures, thereby decreasing the attractiveness of the home to *Ae. aegypti* mosquitoes which favor hot, humid environments. Porous flooring material was only found to decrease dengue risk signiflcantly in univariate analysis in the Indonesia cohort. Porous walls were associated with a decrease which was not statistically signiflcant in either model. Other studies have suggested that housing construction features that affect air flow modulate dengue risk[24, 27]. More research is needed to further evaluate how porous housing materials may impact ventilation and indoor temperature and thus potentially lower mosquito burden.

Our study is limited by arbovirus serology assay accuracy, particularly in the setting of multiple circulating arboviruses. Dengue and Zika are both flaviviruses with consequent potential for cross-reactivity on serology studies. However, since these viruses are transmitted by the same mosquitoes, in places with co-circulation, risk for one virus equates to risk for the other and prior infection with both viruses in some children would be expected. Using validated commercial ELISA kits, we ran all samples in duplicate and found complete concordance between duplicate runs; additionally, positive versus negative results separated clearly when evaluating titer values. We identifled 1 individual in Indonesia and 3 individuals in Fiji who tested positive for Zika and negative for dengue; and the ratio of Zika to dengue positive results in the two countries was dissimilar. Based on these flndings, we infer that at least some, if not all, of our Zika positive results were true positives. Chikungunya virus serology also has the potential to cross-react with other alphaviruses such as Ross River virus. Ross River virus is known to circulate in the broader region but is largely considered to cause asymptomatic infections, so prevalence is not well documented. Given these potential limitations with the Zika and chikungunya serologies and the relatively low prevalence, we elected not to estimate incidence or include them in the risk factor analysis.

For all these viruses, there is also the potential for false positive results due to transfer of maternal antibodies. For this reason, we restricted sampling to children over 6 months of age, past the point of placental antibody transfer and often past the time for exclusive breastfeeding. Additionally, we found that breastfeeding rates at the time of serum sampling was low in both places, that breastfeeding children tested positive and negative on serology, and that breastfeeding was not associated with an increased likelihood of seropositivity. Therefore, we believe our results were not signiflcantly impacted by the possibility of maternal antibody transfer.

One limitation of our incidence estimates is that they are modeled based on seroprevalence and age, not measured by case counts. Unlike an acute febrile surveillance system, serology results cannot tell you when and where a case occurred. However, by testing very young children, we know that infection occurred during their relatively short lifespan and likely around their current residence. The other advantage of this approach to measuring incidence is that we can capture all infections, not only infections that resulted in symptomatic infection and diagnostic testing within a narrow detection window. One caveat though is that children in such a high incidence setting may have had multiple infections with different dengue serotypes, which would be missed by serology, resulting in an underestimate of incidence. However, this problem is more likely as children get older. Additionally, in places where childhood incidence is very high and infection results in lifelong immunity, the incidence in the adult population may be signiflcantly lower than in the child population. Despite these limitations, estimations of recent dengue incidence in children more accurately reflects recent transmission dynamics and can be used to help monitor how disease transmission is changing.

Finally, our risk factor analysis had some limitations. In Fiji we did not identify any built or natural environment risk factors for dengue infection. This may have been due to inadequate statistical power, relatively high or low rates of certain risk factors across the entire study population, or simply the extremely high burden of disease that obscured individual risk factors. A previous study in Fiji during a 2013–2014 dengue outbreak similarly did not identify any demographic or environmental risk factors for infection[44]. In Indonesia where dengue burden was lower and there was greater heterogeneity of key risk factors, such as trash collection, we did flnd that these factors contributed to dengue risk. We elected not to do a combined analysis of the Indonesia and Fiji data given the differences in seropositivity and various risk factors between these two countries. Self-reporting of risk factors, lack of information about neighbor practices that could affect the local environment, and unmeasured housing construction features in neighborhoods based on flood risk (i.e. housing built off the ground in flood-prone areas) are additional limitations of the risk factor analysis. We accounted for unmeasured neighborhood risk factors in our multivariable model by including settlement as a random effect and conducted a secondary analysis of overall settlement flooding and trash collection rates. Improved techniques to objectively measure environmental risk factors such as trash and flood exposure and housing design could improve our understanding of how these factors affect individual risk for arbovirus infection. Despite these limitations, we do see a strong effect of household trash disposal practices on infection risk in Indonesia and indications that flooding and housing construction affects risk and warrants further study.

## Conclusions

In summary, our study found very high rates of dengue in young children living in informal urban settlements in Makassar, Indonesia and Suva, Fiji as well as lower rates of Zika and chikungunya in this population suggesting ongoing low-level transmission of these other two *Ae. aegypti*-transmitted viruses. Household trash collection and community flooding appear to be protective factors against dengue exposure. Further work to evaluate these modiflable risk factors and test interventions designed to disrupt transmission pathways can help mitigate the increasing risk of *Ae. aegypti*-transmitted viruses globally.

## Figures and Tables

**Figure 1:**
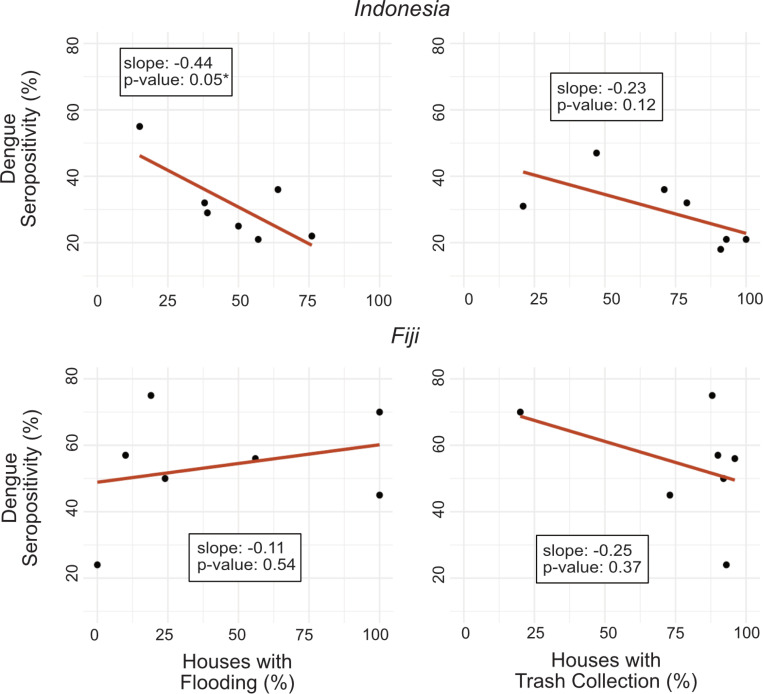
Settlement level dengue seropositivity versus flooding and trash collection rates *Indicates statistically signiflcant result with p-value<0.05.

**Table 1:** Baseline characteristics of children in Fiji and Indonesia

	Fiji n (%) (N = 191)	Indonesia n (%) (N = 181)
*Child characteristics*		
Male	107/191 (56%)	119/181 (66%)
Age: mean (sd)	3.2 (1.2)	3.5 (1.0)
Breastfed in the past 3 months	50/185 (27%)	20/181 (11%)
Breastfeeding currently	35/185 (19%)	17/181 (9%)
*Ethnicity & religion of household respondent*		
*Ethnicity*		
I-Taukei	168/189 (89%)	
Indo Fijian	11/189 (6%)	
Other Fijian or Mixed	10/189 (5%)	
Makassar		108/163 (66%)
Bugis		11/163 (7%)
Toraja		8/163 (5%)
Other Indonesian or Mixed		36/163 (22%)
*Religion*		
Christian	76/189 (40%)	
Lotu Vakarisito	101/189 (53%)	
Other or Mixed (Fiji)	12/189 (6%)	
Islam		152/163 (93%)
Other or Mixed (Indonesia)		11/163 (7%)
*Household characteristics*		
Reports many mosquito bites^1^	156/191 (82%)	151/160 (94%)
Household trash collection^2^	161/191 (84%)	103/160 (64%)
Grows plants^3^	126/191 (66%)	79/160 (49%)
Experiences flooding in or outside the house	52/191 (27%)	86/181 (48%)
Flooring made of porous material^4^	182/191 (95%)	53/160 (33%)
Walls made of porous material^4^	84/191 (44%)	37/160 (23%)
Stores water	145/191 (76%)	146/160 (91%)

Abbreviations: sd = standard deviation

In Fiji, there were 2 children with missing data on ethnicity and religion and 6 children with missing data on breastfeeding status. In Indonesia, there were 21 children with missing data on various household characteristics.

1 Household respondent reported that in the past 6 weeks they experienced mosquitoes biting inside the house at least several times a week.

2 Trash is always collected and taken away or taken to a neighborhood collection point.

3 A household member grows plants in the house, garden, or settlement croplands.

4 Porous housing material included wood, bamboo, woven mat, dirt, and tent material; in contrast, non-porous materials included cement, ceramic tiles, bricks, laminate, granite, and metal.

**Table 2:** IgG Seropositivity of Aedes-transmitted arboviruses in children under 5 years old

Arbovirus	Fiji (N = 191)	Indonesia (N = 181)
*All results*		
Dengue	88 (46.1%)	59 (32.6%)
Chikungunya	5 (2.6%)	5 (2.8%)
Zika	18 (9.4%)	3 (1.7%)
*Multiple infections*		
Dengue & Chikungunya	4 (2.1%)	5 (2.8%)
Dengue & Zika	14 (7.3%)	2 (1.1%)
Dengue & Chikungunya & Zika	1 (0.5%)	0 (0%)

Abbreviations: IgG = Immunoglobulin G.

“All results” includes all children who tested positive on IgG ELISA for each arbovirus, regardless of results of the other serology tests. “Multiple infections” refers to the number of children who tested positive on multiple serology tests, indicating infection with multiple arboviruses during their lifetime, although not necessarily co-infection at the same time.

**Table 3:** Dengue seropositivity by age

Age (years)	Fiji N = 191	Indonesia N = 181
0.5 to < 1^*^	1/10 (10%)	1/3 (33%)
1 to < 2	2/28 (7%)	1/17 (6%)
2 to < 3	10/37 (27%)	9/36 (25%)
3 to < 4	33/57 (58%)	14/58 (24%)
4 to < 5	42/59 (71%)	34/67 (51%)

Dengue risk increases with age in both countries, consistent with continuous increasing exposure over time. This is consistent with endemic transmission rather than a single epidemic.

* Seroprevalence estimates may be artiflcially elevated for this age category because this only includes children > 6 months, not across the entire range. Estimates in this group are also less precise due to small sample size in this group.

**Table 4:** Demographic and environmental risk factors for dengue exposure amongst children in Fiji and Indonesia

	Fiji				Indonesia			
Risk Factors	Negative (N = 103)	Positive (N = 88)	Unadjusted Model OR (95% CI)	Adjusted Model OR (95% CI)	Negative (N = 122)	Positive (N = 59)	Unadjusted Model OR (95% CI)	Adjusted Model OR (95% CI)
Male gender	57 (55%)	50 (57%)	1.1 (0.6–1.9)	0.9 (0.4–1.9)	83 (68%)	36 (61%)	0.7 (0.4–1.4)	0.9 (0.4–2.1)
Age (years)	2.6 (1.2)	3.8 (0.8)	**2.9 (2.1–4.1)** ^*^	**4.0 (2.5–6.3)** ^*^	3.3 (1.0)	3.9 (0.9)	**2.0 (1.4–2.9)** ^*^	**2.2 (1.4–3.6)** ^*^
Breastfed in the past 3 months	33/102 (32%)	17/83 (21%)	0.5 (0.3–1.0)	2.7 (1–7.8)	17 (14%)	3 (5%)	0.3 (0.1–1)	0.7 (0.1–3.9)
Breastfeeding currently	27/102 (26%)	8/83 (10%)	**0.3 (0.1–0.7)** ^*^	1.5 (0.5–4.8)	14 (11%)	3 (5%)	0.4 (0.1–1.3)	0.9 (0.2–4.9)
Reports many mosquito bites^1^	82 (80%)	74 (84%)	1.4 (0.6–2.9)	0.6 (0.2–1.7)	104/111 (94%)	47/49 (96%)	1.6 (0.4–10.9)	3.3 (0.5–21.5)
Household trash collection^2^	86 (83%)	75 (85%)	1.1 (0.5–2.5)	2.2 (0.7–7)	79/111 (71%)	24/49 (49%)	**0.4 (0.2–0.8)** ^*^	**0.3 (0.1–0.8)** ^*^
Grows plants^3^	65 (63%)	61 (69%)	1.3 (0.7–2.4)	1.3 (0.6–3.2)	54/111 (49%)	25/49 (51%)	1.1 (0.6–2.2)	1.1 (0.5–2.4)
Experiences flooding in or outside the house	27 (26%)	25 (28%)	1.1 (0.6–2.1)	0.5 (0.2–1.4)	59 (48%)	27 (46%)	0.9 (0.5–1.7)	1.6 (0.7–3.7)
Flooring made of porous material^4^	97 (94%)	85 (97%)	1.8 (0.4–8.5)	2.5 (0.4–15.3)	43/111 (39%)	10/49 (20%)	**0.4 (0.2–0.9)** ^*^	0.4 (0.2–1.2)
Walls made of porous material^4^	47 (46%)	37 (42%)	0.9 (0.5–1.5)	0.8 (0.3–1.7)	28/111 (25%)	9/49 (18%)	0.7 (0.3–1.5)	1.0 (0.4–2.7)
Stores water	78 (76%)	67 (76%)	1.0 (0.5–2.0)	0.9 (0.4–2.3)	101/111 (92%)	45/49 (90%)	0.8 (0.3–2.7)	0.5 (0.1–2.3)

Abbreviations: OR = odds ratio; CI = confldence interval

* Indicates statistically signiflcant result with a threshold of p < 0.05

Denominators provided for all variables with missing data.

In the adjusted models, we ran a second model for breastfeeding currently that excluded breastfed in the past 3 months and a second model for flooring and walls made of porous material that excluded the individual variables for flooring and walls.

1 Household respondent reported that in the past 6 weeks they experienced mosquitoes biting inside the house at least several times a week.

2 Trash is always collected and taken away or taken to a neighborhood collection point

3 A household member grows plants in the house, garden, or settlement croplands.

4 Porous housing material included wood, bamboo, woven mat, dirt, and tent material; in contrast, non-porous materials included cement, ceramic tiles, bricks, laminate, granite, and metal.

## Availability of data and materials:

At the end of the research trial, deidentifled data will be stored on secure Monash infrastructure and made available, upon application, as approved by the ethics committees. Researchers interested in accessing data may contact the RISE Program (https://doi.org/10.26180/ctjf-vf69)

## Abbreviations

RISE: Revitalizing Informal Settlements and their Environment
SD: standard deviation
IgG: Immunoglobulin G
OR: odds ratio
CI: confldence interval
